# Determinants of drug-related problems among ambulatory type 2 diabetes patients with hypertension comorbidity in Southwest Ethiopia: a prospective cross sectional study

**DOI:** 10.1186/s13104-018-3785-8

**Published:** 2018-09-24

**Authors:** Mohammed Yimama, Habtemu Jarso, Tigestu Alemu Desse

**Affiliations:** 1 Department of Pharmacy, College of Health Sciences, Mizan Tepi University, Mizan, Ethiopia; 2 Department of Epidemiology, College of Health Sciences, Jimma University, Jimma, Ethiopia; 3 Department of Pharmacology and Clinical Pharmacy, School of Pharmacy, College of Health Sciences, Addis Ababa University, Addis Ababa, Ethiopia

**Keywords:** Type 2 diabetes, Hypertension, Drug-related problems, Ethiopia

## Abstract

**Objective:**

The aim of this study was to assess drug-related problems and its determinants in type 2 diabetes patients with hypertension co-morbidity.

**Results:**

A total of 300 type 2 diabetes patients with hypertension co-morbidity were studied. The majority of participants, 194 (64.7%), were males. Mean age of the participants was 54.44 ± 11.68 years. The mean durations of diabetes and hypertension were 5.37 ± 4.79 and 5.15 ± 4.65 years respectively. The most commonly prescribed antidiabetic medications were metformin in 200 (66.7%) and insulin 126 (42%) of the participants. Enalapril was the most commonly prescribed antihypertensive medication; 272 (90.7%). Aspirin was prescribed to 182 (60.7%) participants. Statins were prescribed to one-third (65.67%) of the participants. Eighty-five (28.3%) participants had diabetes related complications other than hypertension. A total of 494 drug related problems were identified. The mean number of drug related problems was 1.65 ± 1.05. The most common drug related problems were need for additional drug therapy (29.35%), ineffective drug (27.94%) and dose too low (15.8%). Independent predictors of drug related problems were age 41–60 years (AOR = 6.87, 95% CI 2.63–17.93), age > 60 years (AOR = 5.85, 95% CI 2.15–15.93) and the presence of comorbidity (AOR = 3.0, 95% CI 1.11–8.16).

## Introduction

Uncontrolled diabetes is the major cause of microvascular and microvascular complications [1–4] and death [5, 6]. The presence of hypertension with diabetes increases risk of mortality by 7.2 fold [7–9] with a greater risk of death in developing nations [10].

Achieving target blood glucose in type 2 diabetes patients with hypertension remains a big challenge despite the availability of different classes of drugs [11]. Studies show that about two-third of type 2 diabetes patients with hypertension fail to achieve target blood glucose and blood pressure [12, 13]. A study in Canada on type 2 diabetes patients with hypertension revealed that 70% of patients receiving mono-therapy did not achieve target blood pressure (BP) whereas the level of uncontrolled BP in patients receiving dual, triple, and 4 medications was 65, 66, and 46% respectively [14].

Multiple medical conditions have been shown to contribute to drug related problems (DRPs) [15–19]. Similarly, type 2 diabetes patients with hypertension often use multiple medications and this may lead to the occurrence of drug related problems [11, 20, 21]. Studies show that the number of DRPs per patient linearly increases with the increase in the number of drugs used [22–25].

Drug related problems may lead to increased morbidity, mortality, healthcare costs, and recurrent hospital admissions and prolonged hospitalization [26]. Cost of drug-related problem related morbidity and mortality exceeds the cost of the medications themselves [27].

In Ethiopia [28] hypertension is the common comorbidity in type 2 diabetes patients. Despite the high prevalence of type 2 diabetes with hypertension and drug treatment related problems, no adequate studies have been conducted in the country. Therefore, this study aimed to investigate the magnitude of DRPS and contributing factors in type 2 diabetes patients with hypertension in a resource limited setting.

## Main text

### Research methods and patients

This was a prospective cross sectional study among type 2 diabetes patients with hypertension comorbidity at Jimma University Specialized Hospital (JUSH) from April 4 to May 11, 2016. The hospital is the only teaching and referral hospital in Southwestern part of Ethiopia. It provides specialized health services for approximately 15,000 inpatient, 160,000 outpatient attendants a year [29]. The hospital ambulatory care clinic serves for 1700 type 2 diabetes and 2017 hypertensive patients. Patients with diabetes and hypertension have regular follow up every month with possible extension to every 3 months for some patients.

The study was approved by institutional review board of Jimma University. We obtained written informed consent from study participants. Sample size was calculated using a single proportion formula with the assumption of 5% margin of error, 95% confidence interval and 50% prevalence of drug related problems (DRPs) in type 2 diabetes patients with hypertension. Through calculation, the total sample size was 309. All type 2 diabetes patients with hypertension who fulfilled the inclusion criteria and who visited diabetes clinic during the study period were included. To ensure the quality of the data; we gave training to data collectors and English version of the data collection questionnaire was translated to Afan Oromo and Amharic and back translated to English. The data collection tool was also pretested. The main outcome of this study was the presence of drug related problems among type 2 diabetes patients with hypertension co-morbidity.

We included patients: age ≥ 18 years old, type 2 diabetes with hypertension, and patients on treatment with anti-diabetic and anti-hypertensive medications for at least 6 months. Participants not willing to participate, with psychiatric comorbidity, and with incomplete medical records were excluded from the study.

We used data abstraction format to collect data on diagnosis, duration of illness, dosage regimen, adverse drug reactions (ADRs), diabetes complications, blood glucose and blood pressure measurements, and laboratory results. Structured questionnaire was used to collect patients’ demographics, and patients’ medication experience. Patients whith evidence of circumstances or events related to drug therapy that actually or potentially interfere with desired health outcomes were considered to have DRP. Drug related problems (DRPs) and possible causes of DRPs were identified using Cipolle’s drug related problem identification tool [8, 15] pharmacotherapy text book and Ethiopian standard treatment guideline [30, 31]. Naranjo Adverse Drug Reaction Probability Scale [32] was to identify ADRs. Adverse Drug Reaction Probability Scale was categorized by taking sum of 10 questions and grouped as definite, probable, possible or doubtful, if the total score is > 9, 5–8, 1–4 and 0 respectively.

Data were collected by trained data collectors (two Pharmacists and one nurse). The data abstracted from patient charts were: diagnoses, comorbidities, dosage regimen, diabetes related complications, laboratory tests, blood glucose and blood pressure measurements of the last three consecutive months.

#### Statistical analysis

Data were analyzed using SPSS Version 21.0 (Chicago, SPSS Inc.). We performed univariate logistic regression tests to assess the association between independent variables and dug related problems (DRPs). Variables with P < 0.25 on a univariate analysis were entered into a multivariable analysis to identify independent predictors of DRPS. Statistical significance was declared for variables with P ≤ 0.05.

#### Operational definitions and definition of terms

*Adverse drug reaction*: any response to a drug which is noxious and unintended and that occurs at normal therapeutic dose [33].

*Dosage regimen*: dose of the medication, frequency of administration, and duration of treatment [3].

*Comorbidity*: the presence of additional diseases in relation to an index disease [34].

*Drug-related problems*: events or circumstances involving drug therapy that actually or potentially interfere with desired health outcomes [25].

*Renal impairment*: the presence of chronic kidney disease, chronic interstitial nephritis, chronic glomerulonephritis, creatinine clearance of less than 50 mL/min, or diabetic nephropathy [35].

*Poly-pharmacy*: consumption of five or more medications [19].

*Concomitant medications*: drugs in which their indications are neither hypertension nor diabetes [36].

### Results

#### Characteristics of participants

Out of 309 participants, 300 (97.08%) of them fulfilled the inclusion criteria and were included in the study. The mean age of the participants was 54.44 ± 11.68 years (range 22–90 years). One hundred ninety-four (64.7%) participants were males. The majority, 87.3%, of the participants were married, 7.67% widowed and 5% divorced. One hundred twenty-six (42%) of the participants were illiterate and over one-third of the participants (34%) were farmers. The mean duration of diabetes was 5.4 ± 4.8 years. The mean duration of hypertension was 5.2 ± 4.7 years. Most of the study participants were diagnosed in the last 5 years.

Seventy (23.3%) participants had at least one co-morbidity. Peptic ulcer disease; 28 (40.00%) and congestive heart failure; 19 (27.14%) were the most common comorbidities. Eighty-five (28.33%) participants had at least one diabetes related complication. Neuropathy was the most common microvascular type 2 diabetes complication; 43 (50.59%) followed by retinopathy; 27 (31.77%).

#### Prescribed Medications

The mean number of prescribed medications was 4.08 ± 1.15 ranging from 2 to 10 medications per patient (Table 1). One hundred and two (34%) of the participants were taking ≥ 5 Medications. Nearly two-third (63.0%) of the participants was on diabetes monotherapy. Metformin was the most common prescribed anti-diabetic medication as monotherapy; 89 (29.67%), followed by insulin; 79 (26.33%). The most common combination therapy was a combination of metformin and glibenclamide; 74 (24.67%).

**Table 1:** Medications prescribed to diabetes patients with hypertension at Jimma University Specialized Hospital

Variable category	Frequency (%)
Number of medications	
< 5	198 (66.00)
≥ 5	102 (34.00)
Type of medication	
Metformin	89 (29.67)
Insulin	79 (26.33)
Glibenclamide	11 (3.67)
Aspirin	182 (60.67)
Lovastatin	84 (28.00)
Simvastatin	85 (28.33)
Atorvastatin	28 (9.33)
Enalapril	237 (79.00)
Hydrochlorothiazide	17 (5.67)
Amlodipine	5 (1.67)
Atenolol	16 (5.33)
Amytriptyline	31 (10.33)
Omeprazole	32 (10.67)
Furosemide	8 (2.67)
Others*	13 (4.33)
Combined anti-diabetic medications	
Metformin + glibenclamide	74 (24.67)
Metformin + insulin	37 (12.33)
Glibenclamide + insulin	7 (2.33)
Metformin + glibenclamide + insulin	3 (1.00)
Combined anti-hypertensive medications	
Enalapril + amlodipine	22 (7.33)
Enalapril + hydrochlorothiazide	13 (4.33)
Hydrochlorothiazide + amlodipine	2 (.67)

*Metoprolol, Salbutamol, Alfuzosin, Carbamazepine, Ranitidine

Enalapril was the most frequently used antihypertensive medication; 237 (79.0%). Hydrochlorothiazide was prescribed for 17 (5.67%) of the participants. A combination of enalapril and amlodipine were most commonly used antihypertensive medications; 22 (7.33%). Aspirin, 182 (60.67%), and Simvastatin, 85 (28.33%), were the most commonly prescribed medications to prevent cardiovascular comorbidities. Thirty-two (10.67%) participants were using omeprazole.

#### Drug related problems and predictors of drug related problems

A total number of 494 drug related problems (DRPs) were identified. The mean number of DRPs was 1.65 ± 1.05 (Table 2). The majority (82%) of the participants had at least one DRP. One hundred twenty-one (40.3%) of the participants had two drug related problems. Most common DRP was noted in participants 41–60 years. Need for additional drug therapy was the most common drug related problem; 29.35% followed by ineffective drug therapy; (27.94%) and dose too low; (15.80%). On adjusted bi-variable logistic regression analysis, independent predictors of drug related problem were age 41–60 years, age > 60 years and the presence of comorbidity (Table 3).

**Table 2:** Drug related problems and causes in type 2 diabetes patients with hypertension

Variable category	Frequency (%)
Number of DRPs	
One	64 (21.3)
Two	121 (40.3)
Three	55 (18.3)
Four	6 (2.0)
Category of DRP and its causes	
Need for additional drug therapy	145 (29.4)
Medical condition requires the initiation of drug therapy	4 (2.8)
Preventive drug therapy required to reduce the risk of developing a new condition	124 (85.5)
Medical condition requires additional pharmacotherapy to attain synergistic or additive effects	12 (8.3)
Required preventive drug therapy to reduce the risk of developing a new condition & a medical condition requires additional pharmacotherapy to attain synergistic or additive effects	5 (3.4)
Ineffective drug	138 (27.9)
The drug not the most effective for the medical problem	114 (82.6)
The drug product is not effective for the indication being treated	24 (17.4)
Dosage too low	78 (15.8)
The dose is too low to produce the desired response	76 (97.4)
Drug interaction reduces the amount of active drug available	2 (2.6)
Noncompliance	60 (12.2)
The patient prefers not to take the medication	6 (10.0)
The patient forgets to take the medication	27 (45.0)
The drug product too expensive for the patient	3 (5.0)
The patient prefers not to take the medication & the patient forgets to take the medication	17 (28.3)
The patient forgets to take the medication & the drug product too expensive for the patient	5 (8.3)
The patient prefers not to take the medication, the drug product too expensive for the patient & the patient forgets to take the medication	2 (3.3)
Unnecessary drug therapy	51 (10.3)
No valid medical indication for the drug	45 (88.2)
Multiple drug products are being used for a condition that requires single drug therapy	4 (7.8)
No valid medical indication for the drug & multiple drug products are being used for a condition that requires single drug therapy	2 (3.9)
Adverse drug reaction	13 (2.6)
The drug product causes an undesirable reaction that is not dose-related	7 (53.9)
The drug product contraindicated due to risk factors	6 (46.2)
Dosage too high	9 (1.8)
Dose too high	7 (77.8)
A drug interaction occurs resulting in a toxic reaction to the drug product	2 (22.2)

**Table 3:** Predictors of drug related problems in type 2 diabetes patients with hypertension

Variable category	DRP		COR (95% CI)	AOR (95% CI)
	Yes	No		
Age in years				
21–40	13	14	1	1
41–60	140	21	7.2 (2.97–17.4)	6.54 (2.58–16.61)*
> 60	93	19	5.27 (2.14–13.00)	5.1 (1.95–13.3)*
Duration of DM in years				
≤ 5	170	31	1	1
6–10	49	12	0.75 (0.36–1.56)	0.87 (0.36–2.1)
≥ 11	27	11	0.45 (0.20–0.9)	0.59 (0.2–1.8)
Duration of HTN in years				
≤ 5	180	32	1	1
6–10	44	13	0.60 (0.3–1.2)	0.7 (0.28–1.65)
≥ 11	22	9	0.44 (0.18–1.03)	0.60 (0.19–1.1)
Comorbid disease				
Yes	65	5	3.52 (1.34–9.22)	3.01 (1.11–8.16)*
No	182	48		1

* Statistically Significant

*DM* diabetes mellitus, *HTN* hypertension, *COR* crude odds ratio, *AOR* adjusted odds ratio

## Discussion

Our study revealed that 82% of the participants had at least one drug related problem. The mean number of drug related problems was 1.65 ± 1.05 per patient. The most common drug related problems were need for additional drug therapy (29.35%), ineffective drug therapy (27.94%) and dose too low (15.8%).

The finding in our set up that 82% of the participants had at least one DRP is comparable with the report by Haugbolle et al. [37] where 80.7% of patients had at least one drug related problem. Similar findings were reported in Adama, Ethiopia [38]. The rate of DRPs in our set up is higher than the findings from India [39] where 71.1% of the patients had at least one drug related problem (mean 1.30 ± 1.10). The higher level of DRPS in our set up may be attributed to the difference in the study populations between ours and the Indians. For example, in the Indian study, all the studied patients did not have hypertension comorbidity. The difference in the study design (prospective interventional study design in Indian Study) and level of care given to diabetes patients might also have contributed for this variation.

The rate of DRP in our study is lower than the findings in Malaysia [19]. This discrepancy might be due to differences in the tool used to assess DRPS and different classification systems of DRPS. For instance, insufficient awareness of health and diseases (22% of all the cases) was considered as DRP in Malay study. In addition, subject variability and difference in level of care given might have contributed for this disparity. In our study, 42.0% of participants were illiterate and patients are often given care by medical interns.

Need for additional drug therapy (29.35%) and ineffective drug therapy (27.94%) were the most common drug related problems observed in our study. The majority (71%) need for statin therapy. Our report is higher than the reports from Malaysia [19], India and Indonesia ([18, 40] which ranged from 4.5 to 15.61%. These variations may be attributed to differences in the tools used to classify drug related problems and study designs. Pharmaceutical Care Network Europe classification of DRPs was used to categorize DRPs in other studies while we used Cipoll’s drug related problem identification tool in our study. Differences in type 2 diabetes management and the study settings might also be the possible reasons. For example, nearly 10% of type 2 diabetes patients with hypertension comorbidity in our set up received Hydrochlorothiazide or Amilodipine while guidelines recommend the use of angiotensin converting enzyme inhibitors, angiotensin receptor blockers or nondihydropyridine clacium channel blockers [41]. The rate of ineffective drug therapy in our study is lower than the study in Indonesia [42] where ineffective drug therapy was identified in 50% of the participants.

Dose too low constituted 15.80% of the total drug related DRPs. This was in line with the study in Thailand [43] and Ambo [44], Ethiopia, but higher than the reports from Malaysia, Indonesia and India; range from 1.3 to 12.92% [16, 33, 34]. This discrepancy may be due to the fact that all of the studies were done in hospitalized patients where there may be better care and nurse supervised medication administration. Differences in classification systems of DRPs may also contribute to such difference.

Noncompliance to medications contributed to 12.15% of the total DRPS. Our result was comparable to Malay [19], but lower than Indian [18] and Nigerian findings [45]. The reason for lower or comparable level of noncompliance in our study is unjustifiable. Unnecessary drug therapy constituted 10.32% of all DRPs. This is higher than the Malay [19] finding, but lower than the Indonesian finding [42]. This may be due to differences in DRP assessment tools, study settings and patient characteristics.

Adverse drug reaction and dosage too high were less frequently occurring DRPs in this study. Age 41–60 years, age > 60 years and the presence of comorbidity were independent predictors of drug related problems.

## Conclusions

The rate of drug related problems in type 2 diabetes patients with hypertension is high. The most common DRPs were need for additional drug therapy, ineffective drug therapy and dose too low. The most common causes of DRPs were need for preventive drug therapy and the drug not the most effective for the medical problem. Age 41–60 years, age > 60 years and the presence of comorbidity were independent predictors of drug related problems.

## Limitations

The limitation of this study was the small sample size to generalize the findings to the general population.

## Abbreviations

DRPs: drug related problems
T 2 DM: type two diabetes mellitus
COR: crude odds ratio
AOR: adjusted odds ratio
JUSH: Jimma University Specialized Hospital
ADR: adverse drug reaction
